# Effects of Different Cropping Patterns on Soil Microbial Community and Function in Ningxia Irrigation-Silted Soil

**DOI:** 10.3390/microorganisms14051089

**Published:** 2026-05-11

**Authors:** Baiyun Li, Qing Zhao, Hongna Li, Zehua Xu, Tao Zhou, Xinnian Guo, Lina Zhou

**Affiliations:** 1 Horticultural Research Institute, Ningxia Academy of Agriculture and Forestry Sciences, Yinchuan 750002, China; lby713snn@163.com (B.L.); xzh850925@126.com (Z.X.); 2 Institute of Environment and Sustainable Development in Agriculture, Chinese Academy of Agricultural Sciences, Beijing 100081, China; zhaoqing03@caas.cn (Q.Z.); lihongna@caas.cn (H.L.); 3 Agricultural Resources and Environment Institute, Ningxia Academy of Agriculture and Forestry Sciences, Yinchuan 750002, China; zhoutao6084609@163.com (T.Z.); gxnian@163.com (X.G.)

**Keywords:** crop rotation, soil bacterial community, soil fungal community, microbial diversity, functional prediction, sustainable agriculture

## Abstract

Irrigation-silted soil in Ningxia represents a unique, anthropogenically modified agroecosystem, beneficial for regional food security. Yet, how different agricultural management techniques influence soil microbiome diversity remains poorly explored. Full-length amplicon sequencing (16S rRNA and ITS) was applied to assess the effects of vegetable and maize cultivation, relative to an uncultivated wasteland control, on soil bacterial and fungal community. Cropping patterns significantly influenced microbial alpha diversity, with contrasting effects on bacterial and fungal communities. Specifically, bacterial diversity peaked in vegetable fields, while fungal diversity was highest in maize fields. Both the bacterial and fungal community structures differed markedly among the three land-use types (*p* < 0.01). Although *Pseudomonadota* (among bacteria) and *Ascomycota* (among fungi) were the dominant phyla across all soils, each land-use type harbored distinct biomarkers. For example, vegetable fields facilitated the enrichment of the genus *Fusarium*, whereas maize fields were characterized by both *Pseudomonadota* and diverse saprotrophic fungi. Based on functional prediction, sulfur oxidation and cellulose decomposition were enhanced in soil with vegetable cultivation, while maize cultivation promoted relatively broader metabolic activity and enriched arbuscular mycorrhizal fungi compared with the control. Agricultural practices act as selective filters shaping soil microbial assembly and function, which provide a theoretical foundation for sustainable management strategies aimed at preserving soil health.

## 1. Introduction

Irrigation-silted soil in Ningxia, formed through long-term irrigation, siltation, cultivation, and fertilization, represents a unique soil type in the Yellow River irrigation district [1,2]. As an anthropogenically modified agroecosystem distributed across alluvial plains and surrounding farmlands, this soil exhibits distinctive formation processes and high fertility, rendering it essential for agricultural development in this arid region. And, by extension, making its sustainable management fundamental for maintaining regional food security and ecological stability [3,4].

Soil microorganisms play a central role in ecosystem functioning, driving organic matter decomposition, nutrient cycling, and soil structure formation [5]. Cropping patterns strongly influence these communities by modifying soil physicochemical properties, root exudates, and crop residues [6,7]. However, the microbial responses to different cropping systems in Ningxia’s irrigation-silted soil remain poorly understood. This knowledge gap is particularly relevant given the rapid expansion of vegetable cultivation for export alongside the region’s long tradition of maize farming. Understanding how vegetable versus maize cultivation alters microbial community structure and function will provide critical insights into soil ecosystem processes and inform the sustainable management of irrigation-silted soils.

To address this gap, we investigated microbial community structure and function in irrigation-silted soils under three land-use types: uncultivated wasteland (CK), vegetable fields planted with choy sum (CD), and maize fields planted with Denghai 605 (YM). Using full-length 16S rRNA and ITS sequencing, which enables higher-resolution taxonomic annotation than conventional short-read methods, we compared the bacterial and fungal community composition and predicted ecological functions across these systems. We hypothesized that distinct land-use types exert specific selective pressures on the soil environment, thereby shaping unique microbial community structures and functional profiles. Specifically, we posited that intensive vegetable cultivation might enrich specific copiotrophic or pathogenic taxa due to high nutrient inputs, whereas maize cultivation would foster beneficial symbiotic networks. Our goal was to elucidate how different cropping patterns shape soil microbial communities and to provide a theoretical basis for the sustainable management and utilization of irrigation-silted soils.

## 2. Materials and Methods

### 2.1. Study Area Description

The study area is located in the Yellow River irrigation district of Ningxia Hui Autonomous Region, characterized by an arid continental climate at an elevation of 1100 m. The region experiences annual precipitation of 180 mm with an evaporation rate of 1200 mm, with an annual mean temperature of 9 °C, 2950 h of sunshine, and a frost-free period of 164 days. This area represents a typical distribution zone of irrigation-silted soil, which has been formed through artificial Yellow River irrigation and long-term agricultural cultivation processes.

### 2.2. Sample Collection and Processing

Vegetable and maize plots were established in April 2024 and harvested in September 2024 under local conventional management practices. Three land-use types were included: uncultivated wasteland (CK), vegetable fields planted with choy sum for Hong Kong export (CD), and maize fields planted with Denghai 605 (YM). Vegetable fields (CD) are mainly treated with organic fertilizer (6 t/ha) and nitrogen phosphorus potassium (2:1:1.2) compound fertilizer (0.7 t/ha), while corn fields (YM) are managed with conventional chemical fertilizers (N 375 kg/ha, P_2_O_5_ 150 kg/ha, K_2_O 75 kg/ha). On 5 September 2024, after crop harvest, soil samples were collected from the 0–20 cm layer using a diagonal five-point sampling method.

For each land-use type, three spatially independent replicate plots (each 20 × 20 m) were established, with a minimum distance of 1 km between plots to ensure spatial independence and avoid pseudoreplication. The collected soil was homogenized, and approximately 1 kg was retained using the quartering method. Samples were placed in sterile bags, transported on ice, and processed under sterile conditions. Each sample was divided into two portions: one portion was air-dried and sieved for physicochemical analysis, and the other was stored at −80 °C for DNA extraction and microbial community analysis.

### 2.3. Soil Physicochemical Properties Investigation

Soil moisture content was measured gravimetrically by drying fresh soil samples to a constant weight at 105 °C for 24 h. Soil properties were determined following standard protocols. Soil pH was measured by the potentiometric method with a soil-to-water ratio of 1:2.5. Electrical conductivity (EC) was determined using a conductivity meter (1:5 soil-to-water ratio). Soil organic matter was analyzed using the potassium dichromate external heating method. Total nitrogen (TN) was measured by the Kjeldahl method. Total phosphorus (TP) was determined by the NaOH fusion–molybdenum antimony colorimetric method, and total potassium (TK) by NaOH fusion–flame photometry. Available nitrogen (AN) was measured using the alkali hydrolysis diffusion method. Available phosphorus (AP) was measured using extraction with 0.5 mol/L NaHCO_3_ followed by molybdenum antimony colorimetry. Available potassium (AK) was determined using extraction with 1 mol/L NH_4_OAc followed by flame photometry. Total salt content was measured using the residue drying method.

### 2.4. DNA Extraction and PCR Amplification

Total soil DNA was extracted using the TGuide S96 magnetic bead soil/fecal genomic DNA extraction kit (TIANGEN Biotech, Beijing, China). The concentration and purity of the extracted DNA were determined using a Qubit 2.0 Fluorometer (Thermo Fisher Scientific, Waltham, MA, USA) with the Qubit TMdsDNA HS Assay Kit. Additionally, DNA integrity was verified by 1% agarose gel electrophoresis. Only DNA samples with a concentration > 1 ng/μL and no obvious degradation bands were subjected to subsequent full-length PCR amplification and library construction. Full-length bacterial 16S rRNA genes were PCR-amplified using primers F (5′-AGRGTTTGATYNTGGCTCAG-3′) and R (5′-TASGGHTACCTTGTTASGACTT-3′). Full-length fungal ITS genes were PCR-amplified using primers F (5′-CTTGGTCATTTAGAGGAAGTAA-3′) and R (5′-TCCTCCGCTTATTGATATGC-3′). Full-length 16S rRNA and ITS primers were selected to sequence the nearly complete target genes (~1.5 kb for bacteria and the entire ITS region for fungi). Compared to conventional short-read sequencing (which targets only partial regions like V3–V4 or ITS1/2), this approach significantly enhances taxonomic resolution and assignment accuracy, enabling a more reliable identification of key microbial taxa at the genus level. The PCR reaction system was 30 μL, containing 15 μL KOD ONE MM premix, 1.5 μL forward primer (10 μmol/L), 1.5 μL reverse primer (10 μmol/L), 1.5 μL template DNA, and 10.5 μL sterile water. The PCR amplification program was 95 °C pre-denaturation for 5 min; 25 cycles of 95 °C denaturation for 10 s, 55 °C annealing for 30 s, 72 °C extension for 90 s; final extension at 72 °C for 2 min.

### 2.5. High-Throughput Sequencing and Data Analysis

After purification, PCR products were sequenced using the PacBio platform (PacBio Sequel II, Pacific Biosciences, Menlo Park, CA, USA). Lima v1.7.0 software was used to identify Circular Consensus Sequencing (CCS) through barcodes to obtain Raw-CCS sequence data. Cutadapt v1.9.1 software was used to identify and remove primer sequences, followed by length filtering to obtain Clean-CCS sequences without primer sequences. UCHIME v4.2 software was used to identify and remove chimeric sequences, resulting in Effective-CCS sequences. Effective CCS sequences were clustered into operational taxonomic units (OTUs) at 97% similarity level. Species annotation was performed using the Silva database (release 138.1) for bacteria and UNITE database (version 9.0) for fungi. To ensure comparability across samples with varying sequencing depths, the original OTU abundance table was rarefied (subsampled) to the minimum number of sequences observed in a single sample prior to alpha diversity analysis. Alpha diversity indices (Chao1 and Shannon indices) were calculated using the vegan package (version 2.6-8) in R (version 4.1.3). For β-diversity and community composition analyses, the OTU counts were transformed into relative abundances to standardize the data. Principal coordinate analysis (PCoA) based on the Bray–Curtis similarity matrix at ASV level was performed for β-diversity analysis. FAPROTAX [8] and FUNGuild [9] were used for bacterial and fungal ecological functional prediction, respectively.

### 2.6. Statistical Analysis

The Kruskal–Wallis test was performed using the vegan package (version 2.6-8) to determine whether there were overall significant differences in α-diversity indices among multiple groups. Pairwise comparisons were conducted using the Wilcoxon rank-sum tests, followed by Bonferroni correction for multiple comparisons. Permutational multivariate analysis of variance (PERMANOVA) was used to test for significant differences in beta diversity among sample groups. LEfSe [10] (version 1.1.2) was used to screen features with significant differences through the non-parametric Kruskal–Wallis rank sum test (*p* < 0.05), and linear discriminant analysis (LDA) was utilized to evaluate the effect size of each feature (LDA score > 4). Redundancy analysis (RDA) was performed using the vegan package (version 2.6-8) in R (version 4.1.3) to determine the relationship between soil physicochemical properties and microbial community structure.

## 3. Results

### 3.1. Soil Physicochemical Properties

There were significant differences in soil physicochemical properties among the three land-use types (Table 1). Organic matter content was highest in vegetable fields (22.0 g/kg), which was more than double the amount in wasteland (10.2 g/kg) and 41.0% higher than in maize fields (15.6 g/kg). Nutrient contents generally exhibited the following order: vegetable field > maize field > wasteland. For example, the TN contents in CD, YM, and CK soils were 1.72, 1.24, and 0.81 g/kg, respectively, while corresponding AN values were 163, 158, and 26 mg/kg. The significantly lower nutrient availability in the wasteland soils likely reflects minimal external nutrient inputs and limited organic matter turnover.

In contrast, the soil salt content showed the opposite trend (CK > YM > CD), with wasteland soils reaching 3.71 g/kg, compared to 0.82 g/kg in maize fields and 0.76 g/kg in vegetable fields. This accumulation of soluble salts in wasteland may be attributed to the absence of leaching irrigation practices, which are common in cultivated systems. All soils were weakly alkaline, with pH values ranging from 8.29 to 8.71, indicating no substantial pH shift across land-use types despite notable differences in organic matter and salinity.

### 3.2. Soil Microbial Community Diversity Under Different Cropping Patterns

**Figure 1:**
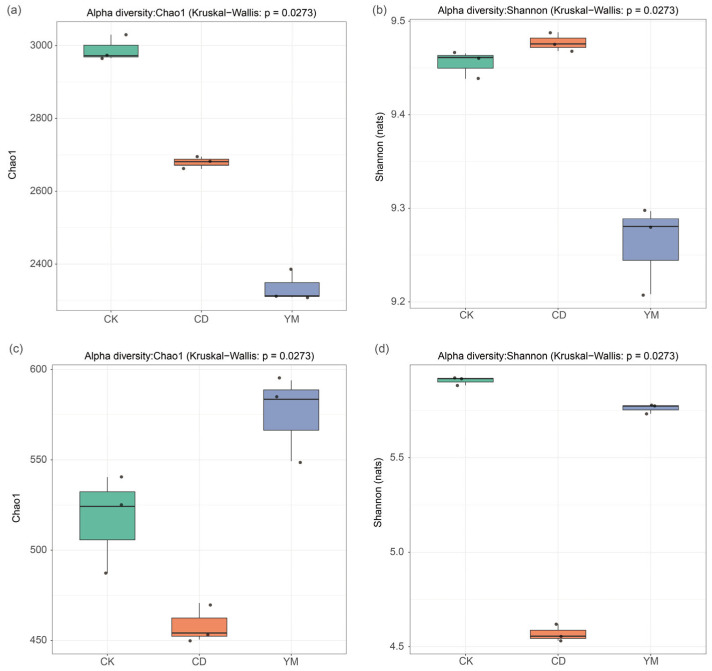
Alpha diversity of bacterial and fungal communities from different land-use types. (**a**) Chao1 index of bacteria; (**b**) Shannon index of bacteria; (**c**) Chao1 index of fungi; (**d**) Shannon index of fungi. CK: abandoned land; CD: vegetable land (choy sum for Hong Kong); YM: maize land (Denghai 605).

For the bacterial communities, the effective sequences per sample ranged from 44,652 to 52,213 (average length ~1450 bp). For the fungal communities, the sequences per sample ranged from 43,033 to 59,440 (average length ~590 bp). To ensure fair comparisons for downstream alpha diversity analyses, the bacterial and fungal OTU tables were rarefied to uniform depths of 44,652 and 43,033 sequences per sample, respectively. To investigate the effects of different cropping patterns on soil microbial diversity, we conducted α-diversity analysis on soil samples collected from the wasteland (CK), vegetable field (CD), and maize field (YM). Microbial diversity was assessed by simultaneously measuring the Chao1 and Shannon indices of the bacterial (16S rRNA gene) and fungal (ITS gene) communities (Figure 1).

The Kruskal–Wallis tests revealed significant differences in both bacterial and fungal α-diversity among different cropping patterns (*p* < 0.05). However, in pairwise comparison analysis, the Wilcoxon rank-sum tests with the more stringent Bonferroni correction failed to identify significant differences between any two groups (*p* > 0.05), potentially due to the stringent correction and limited sample size.

Nevertheless, from a purely descriptive perspective, we observed that the average Chao1 index, which primarily reflects species richness and is particularly sensitive to low-abundance species, showed a numerical decrease from wasteland to cultivated soils (CK > CD > YM) (Figure 1a). The Shannon diversity index, which considers both species richness and evenness, exhibited a different pattern, with vegetable fields numerically showing the highest diversity (CD > CK > YM) (Figure 1b).

For fungal communities, interestingly, the fungal Chao1 index showed an opposite pattern compared to bacteria, with maize fields exhibiting the highest species richness (YM > CK > CD) (Figure 1c). The fungal Shannon diversity index was similar between wasteland and maize fields, both higher than vegetable fields (Figure 1d).

### 3.3. Soil Microbial Community Composition Under Different Cropping Patterns

#### 3.3.1. Bacterial Community Composition

**Figure 2:**
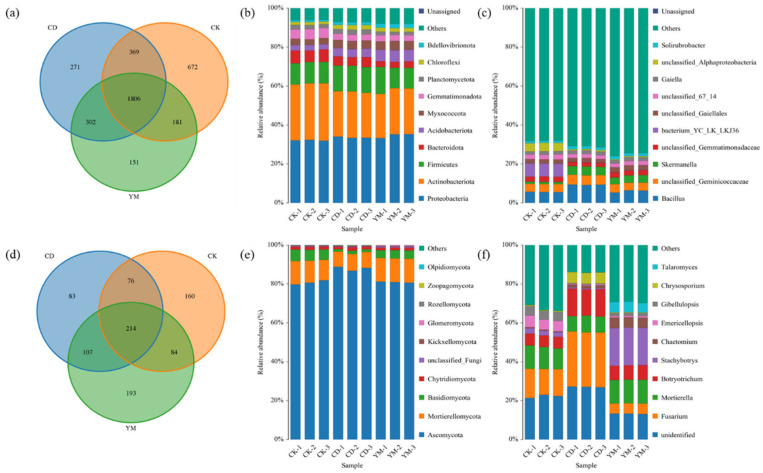
Soil microbial community structure under different land-use types. (**a**) Venn diagram of bacterial OTUs; (**b**) relative abundance of bacterial phyla; (**c**) relative abundance of bacterial genera; (**d**) Venn diagram of fungal OTUs; (**e**) relative abundance of fungal phyla; (**f**) relative abundance of fungal genera. CK: abandoned land; CD: vegetable land (choy sum for Hong Kong); YM: maize land (Denghai 605).

Under different cropping patterns, the bacterial community structures showed both differences and commonalities. As shown in Figure 2a, the number of shared operational taxonomic units (OTUs) among the three land-use types (wasteland CK, vegetable field CD, and maize field YM) was 1806, accounting for 48.13% of the total OTUs. This indicates that nearly half of the bacterial taxa were retained under different land-use patterns. In addition, CK and CD shared 369 unique OTUs, while CK and YM shared 181 unique OTUs, and CD and YM shared 302 unique OTUs. Meanwhile, CK, CD, and YM contained 672, 271, and 151 unique OTUs, respectively. The highest number of unique OTUs in wasteland suggests that undisturbed soils may harbor a greater reservoir of specialist or rare bacterial taxa, which may be lost or suppressed under cultivation.

At the phylum level (Figure 2b), the dominant bacteria detected in all samples mainly included *Pseudomonadota*, *Actinomycetota*, and *Bacillota*. *Pseudomonadota* dominated in all samples with relative abundances of approximately 30–35%, followed by *Actinomycetota* with relative abundances of approximately 20–30%, and *Bacillota* accounting for approximately 10–15% in each sample. These phyla are commonly reported as major components of agricultural soils and are often associated with nutrient cycling, decomposition of organic matter, and adaptation to variable soil environments [11]. This suggests that regardless of land use, soil conditions remain favorable for these copiotrophic bacteria. At the genus level (Figure 2c), differences among samples became more pronounced. Dominant bacterial genera in soil included *Bacillus*, *Skermanella*, unclassified *Gemmatimonadaceae* bacteria, and unclassified *Gaiellales* bacteria. Notably, the relative abundances of Bacillus and *Skermanella* in the vegetable field (CD) and maize field (YM) samples were higher than in the wasteland (CK) samples. This pattern may be related to cultivation-induced changes in soil conditions, as Bacillus are known for their ability to degrade complex organic matter and promote plant growth [12], while *Skermanella* has been associated with rhizosphere environments [13]. Their enrichment in cultivated soils likely reflects the input of crop residues, root exudates, and organic fertilizers, which create favorable conditions for these beneficial bacteria.

#### 3.3.2. Fungal Community Composition

The variation patterns of the fungal communities under different land-use types differed markedly from those of the bacteria. As shown in Figure 2d, the number of shared OTUs among the three land-use types was 214, accounting for 23.34% of the total OTUs, which was much lower than the proportion of shared OTUs in bacterial communities. This indicates that fungal communities are more sensitive to land-use change and cropping patterns. CK, CD, and YM contained 160, 83, and 193 unique OTUs. Interestingly, this indicates that intensive vegetable cultivation strongly reduced unique fungal richness, despite these soils possessing the highest organic matter and nutrient contents (Table 1). In addition, CD and YM shared 107 unique OTUs, which was higher than the 76 OTUs shared between CK and CD and the 84 OTUs shared between CK and YM. This result implies that agricultural practices, despite their differences, impose common selective pressures on fungal communities.

At the phylum level (Figure 2e), Ascomycota was the overwhelmingly dominant phylum in all samples, with relative abundances as high as 80%. This was followed by *Mortierellomycota* and *Basidiomycota*, but their relative abundances were much lower than Ascomycota. The prevalence of Ascomycota is typical in arable soils, as many members of this phylum are saprotrophic or plant-associated and tolerate disturbance well [14]. Notably, the relative abundance of Ascomycota in CD was higher than in CK and YM, possibly due to the continuous input of easily decomposable organic matter (e.g., vegetable residues) that favors fast-growing ascomycete decomposers. At the genus level (Figure 2f), fungal community differences among different land-use types were more pronounced. CD was dominated by Fusarium as the main dominant genus, whereas YM samples were mainly dominated by Stachybotrys. Particularly, the relative abundance of Fusarium in CD was higher than in the other two land-use types. Fusarium includes several species that are important saprophytes and plant pathogens, and its enrichment in CD may be linked to intensive cultivation and continuous nutrient inputs [15]. In contrast, the dominance of Stachybotrys in YM, a genus typically associated with cellulose-rich substrates [16], suggests that maize-associated soils may favor fungal taxa adapted to the decomposition of plant residues or specific substrate conditions.

Overall, these results indicate that fungal communities were more strongly shaped by cropping patterns than bacterial communities, and that land-use change had a more pronounced effect on fungal specificity and genus-level dominance.

#### 3.3.3. Community Structure Difference Analysis

**Figure 3:**
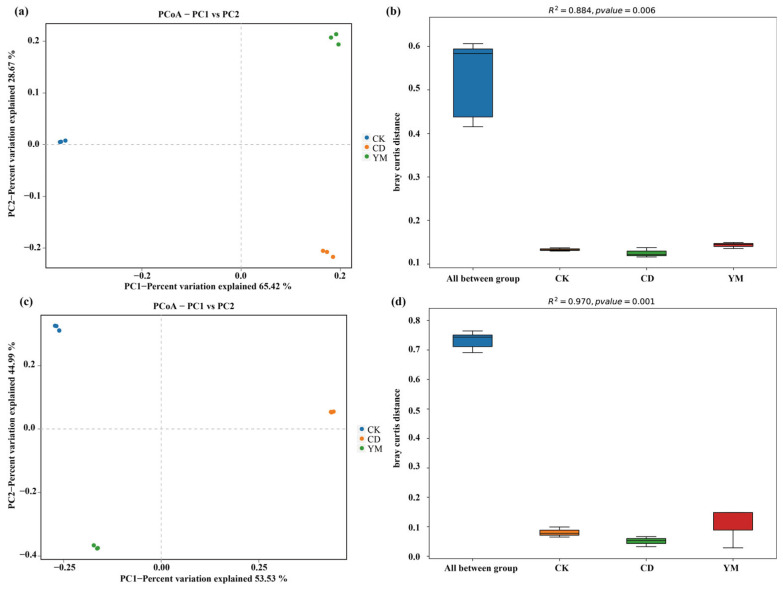
Soil bacterial and fungal community structure and inter-group differences under different land-use types. (**a**) Bacterial community PCoA based on Bray–Curtis distance; (**b**) boxplots of Bray–Curtis distances among bacterial communities and PERMANOVA results (R^2^ = 0.884, *p* = 0.006); (**c**) fungal community PCoA based on Bray–Curtis distance; (**d**) boxplots of Bray–Curtis distances among fungal communities and PERMANOVA results (R^2^ = 0.970, *p* = 0.001). CK: abandoned land; CD: vegetable land (choy sum for Hong Kong); YM: maize land (Denghai 605).

To systematically evaluate structural differences in soil microbial communities, we performed principal coordinate analysis (PCoA) and permutational multivariate analysis of variance (PERMANOVA) based on the Bray–Curtis distance (Figure 3).

The PCoA results for bacterial community structure (Figure 3a) revealed clear separation among the three treatments. PC1 and PC2 explained 65.42% and 28.67% of the community variation, respectively, indicating that the two cultivation methods had significant directional effects on bacterial community structure. The PERMANOVA analysis (Figure 3b) further confirmed that inter-group differences accounted for 88.4% of total variation (R^2^ = 0.884, *p* = 0.006) among the sampled plots. These results demonstrate that the bacterial community composition differed strongly among these specific land-use types, though this high effect size likely reflects the localized sampling scale.

Similarly, the PCoA results for fungal community structure (Figure 3c) showed distinct clustering patterns. PC1 and PC2 explained 53.53% and 44.99% of the community variation, respectively, suggesting that cultivation methods and crop types profoundly affected fungal community structure. The PERMANOVA results (Figure 3d) further confirmed that community differences among different treatment groups could explain 97.0% of the total variation (R^2^ = 0.970) with highly significant differences (*p* = 0.001). Therefore, fungal communities may be rendered more sensitive to land-use conversion compared to bacterial communities.

**Figure 4:**
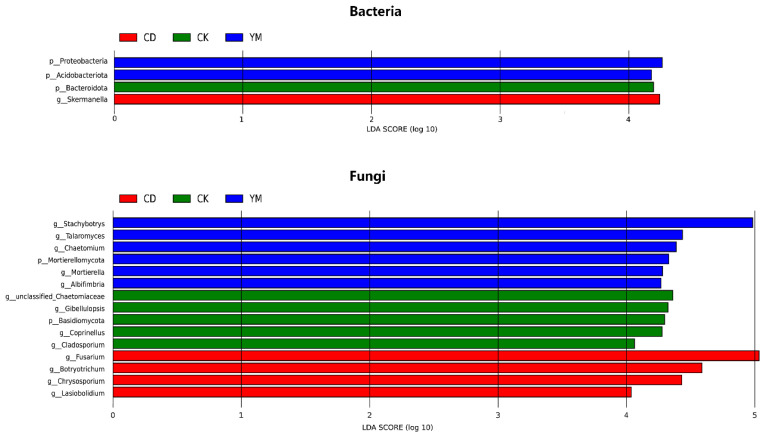
Differential microbial taxa identified by LEfSe under different land-use types.

We further employed LEfSe analysis, which revealed clear and distinct taxonomic signatures for both bacterial and fungal communities (Figure 4).

In the bacterial community, each soil environment harbored a unique set of indicators. The intensively managed vegetable fields (CD) were uniquely characterized by the significant enrichment of the genus *Skermanella*, a diazotrophic bacterium often associated with the rhizosphere of cultivated crops [17]. Maize fields (YM) were distinguished by biomarkers at a higher taxonomic level, specifically the phyla *Pseudomonadota* and *Acidobacteriota*, reflecting their broad metabolic capabilities in carbon and nutrient cycling [18]. The uncultivated wasteland (CK) was defined by biomarkers from the phylum *Bacteroidota*, which are commonly associated with the degradation of complex organic matter in undisturbed soils [19].

A similar pattern of niche-specific enrichment was observed among the fungi. Vegetable cultivation fostered a distinct group of fungal biomarkers, most notably the genera Fusarium, *Botrytichum*, and *Chrysosporium*. The enrichment of Fusarium, which includes various plant pathogens, suggests an elevated risk of soil-borne disease accumulation under conditions of continuous cropping and frequent fertilization [20]. In stark contrast, maize fields supported a broad suite of saprotrophic fungi, with Stachybotrys, *Talaromyces*, *Chaetomium*, and *Mortierella* emerging as the most significant indicators. These genera are known for their capacity to decompose lignocellulosic materials, aligning with the abundance of maize stover residues [21]. Wasteland soil maintained its own unique fungal signature, marked by the phylum Basidiomycota and associated genera like *Gibellulopsis* and *Cladosporium*, which are typical of stable, less disturbed ecosystems [22].

**Figure 5:**
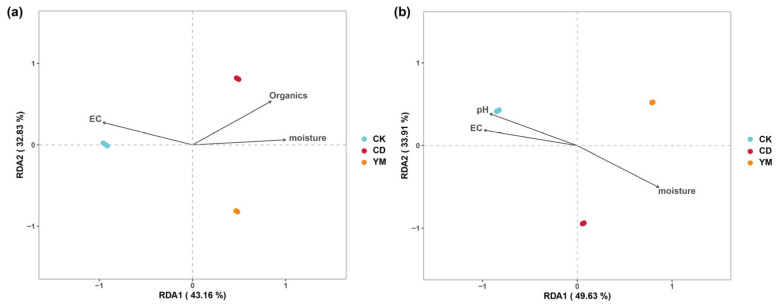
Redundancy analysis (RDA) of microbial community structures as influenced by edaphic variables. (**a**) Redundancy analysis of bacterial communities and soil properties; (**b**) Redundancy analysis of fungal communities and soil properties.

The redundancy analysis (RDA) revealed that selected edaphic variables significantly shaped microbial community structures (Figure 5). For bacteria (Figure 5a, 75.99% of the total variation explained), the vegetable field (CD) communities were strongly positively correlated with organic matter, reflecting intensive fertilization, whereas wasteland (CK) communities were primarily driven by electrical conductivity (EC). Similarly, the fungal communities (Figure 5b, 83.54% explained) in CK were strongly associated with elevated pH and EC. In contrast, cultivated soils (CD and YM) were decoupled from these salinity constraints and aligned more with moisture gradients.

### 3.4. Functional Prediction of Soil Microorganisms Under Different Cropping Patterns

**Figure 6:**
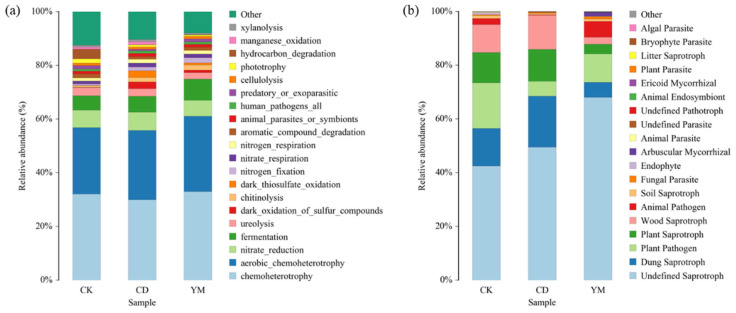
Microbial community function prediction. (**a**) Bacterial ecological functions predicted by FAPROTAX under different cropping practices. (**b**) Composition and relative abundance of assigned fungal functional groups (guild) inferred by FUNGuild.

To infer the functional characteristics of the microbial communities, we predicted the ecological functions of bacteria using FAPROTAX and the functional guilds of fungi using FUNGuild (Figure 6).

For bacteria, the analysis revealed both commonalities and distinct differentiation (Figure 6a). Across all three land-use types, chemoheterotrophy and aerobic chemoheterotrophy were the dominant functions, reflecting the fundamental role of soil bacteria in organic matter decomposition and carbon cycling. Against this shared functional background, each site displayed pronounced specialization. Vegetable fields (CD) showed a distinct advantage in the dark oxidation of sulfur compounds and cellulolysis. These functions are closely linked to the application of sulfur-containing fertilizers and the decomposition of plant residues. In contrast, maize fields (YM) exhibited significantly higher activity in basic metabolic functions like fermentation, indicating active organic matter turnover. Wasteland (CK) was uniquely characterized by a higher potential for hydrocarbon degradation, likely related to the decomposition of natural compounds secreted by wild plants.

A similar pattern was observed for the fungal communities (Figure 6b). Saprotrophic groups were the dominant functional guild in all soils, underscoring the primary role of fungi in decomposition. However, the composition of these guilds varied significantly with land use. Vegetable fields were markedly enriched in dung saprotrophs, a direct reflection of the frequent application of organic fertilizers like manure. The maize fields showed a clear advantage in arbuscular mycorrhizal fungi, highlighting the ecological importance of this plant–fungal symbiosis for maize nutrient acquisition. Interestingly, the relative abundance of plant pathogens was highest in the uncultivated wasteland, which may reflect a natural balance of plant–pathogen interactions in an unmanaged ecosystem.

## 4. Discussion

Different cropping patterns in Ningxia’s irrigation-silted soils acted as strong ecological filters, shaping distinct bacterial and fungal communities with specialized functions [23]. The use of full-length sequencing strengthened the resolution of community profiling compared with conventional short-read methods, enabling more precise interpretation of these shifts.

We observed opposite diversity trends between bacteria and fungi, underscoring their distinct ecological drivers [24]. Vegetable fields promoted higher bacterial evenness, likely due to the diverse carbon inputs from organic fertilizer applications, which create heterogeneous niches despite tillage homogenization [25]. In contrast, maize fields exhibited greater fungal richness, reflecting the strong symbiotic association between maize roots and arbuscular mycorrhizal fungi [26]. This divergence may indicate that bacterial diversity is more responsive to organic matter heterogeneity, whereas fungal diversity may be more closely linked to host plant identity and root-associated interactions. Vegetable fields (CD) contained significantly higher levels of organic matter and nutrients. However, we observed a reduction in unique fungal richness (Figure 2d). This apparent paradox can be attributed to the ‘niche filtering’ effect and competitive exclusion driven by intensive management. High-input fertilization and frequent tillage in CD plots create a highly selective environment. This environment favors fast-growing, opportunistic copiotrophic fungi (e.g., *Fusarium*). These taxa may outcompete and suppress rarer or more specialized fungi. Excessive nutrient availability may inhibit oligotrophic fungi adapted to nutrient-poor conditions. This may further reduce the reservoir of unique fungal taxa.

The LEfSe analysis identified unique microbial signatures under each cropping system. In vegetable soils, enrichment of the diazotrophic genus *Skermanella* likely reflects high nitrogen availability and irrigation-driven anaerobic microsites that support complex nitrogen cycling [13,27]. The maize soils were co-dominated by *Pseudomonadota*, major players in carbon and nitrogen turnover [28,29], and *Acidobacteriota*, which are known specialists in degrading complex organic matter [30], indicating adaptation to crop residue inputs. For fungi, vegetable soils were enriched in Fusarium, a genus containing numerous plant pathogens [31,32]. This signals a potential agronomic risk associated with intensive monoculture. Co-occurring saprotrophic genera such as *Botrytichum* [33] and *Chrysosporium* [34] may contribute to organic matter turnover. By contrast, maize soils supported a suite of cellulose-degrading fungi including *Stachybotrys* [35], *Chaetomium* [36], and *Mortierella* [37], reflecting their role in straw decomposition. *Talaromyces* spp. were also enriched; some members of this genus are known to promote plant nutrition and stress resistance [38], suggesting a potential beneficial role in maize agroecosystems.

The predicted functional profiles revealed clear cropping-related differentiation. Vegetable cultivation favored dung saprotrophs and sulfur-oxidizing bacteria, consistent with manure and sulfur fertilizer inputs. This functional shift is quantitatively supported by our RDA results, which identified organic matter as the primary driver of CD bacterial communities (Figure 5a). Conversely, the strong association of wasteland (CK) microbiomes with elevated EC and pH (Figure 5) highlights the harsh saline–alkaline baseline of unmanaged soils. Thus, agricultural practices fundamentally alleviated these natural edaphic constraints, acting as a deterministic filter that reshaped the soil microbiome. In maize soils, enrichment of arbuscular mycorrhizal fungi emphasized their role in maize nutrient acquisition [39,40]. In wasteland soils, plant pathogens were relatively more abundant, possibly reflecting natural plant–pathogen equilibrium in unmanaged ecosystems. These findings align with previous reports that agricultural inputs selectively filter microbial traits to exploit altered soil environments [41,42]. Regarding plant pathogens, an apparent discrepancy emerged. While FUNGuild predicted the highest ‘Plant Pathogen’ abundance in the uncultivated wasteland (CK) (Figure 6b), the taxonomic analysis firmly identified *Fusarium*—a severe agricultural pathogen—as the defining biomarker for vegetable fields (CD) (Figure 4). This reflects the inherent limitation of database-driven predictive tools, which may broadly categorize indigenous, benign fungi in unmanaged ecosystems as ‘pathogens’. In contrast, the empirical detection and significant enrichment of *Fusarium* in CD indicate a tangible agronomic threat. To fully appreciate the novelty of these shifts, it is crucial to compare our system directly with the recent agroecological literature. Recent studies have frequently reported a general trend of microbial homogenization and increased susceptibility to pathogen invasion under long-term intensive cropping. Directly comparing our findings with these reports reveals a unique, divergent successional trajectory specific to irrigation-silted soils. Unlike typical agricultural soils that degrade from a rich natural baseline, our unmanaged baseline (CK) was a harsh saline–alkaline environment. Consequently, cultivation did not universally degrade the microbiome. Instead, we observed a strict crop-dependent divergence: while intensive vegetable farming (CD) aligned with recent reports by escalating pathogenic risks (*Fusarium*), maize cultivation (YM) acted as an ecological ameliorator by alleviating salinity constraints and promoting beneficial symbionts. This specific contrasting dynamic is a unique contribution that extends our current understanding of *Anthrosol microbiomes* [43,44].

Several limitations should be acknowledged. This study is based on a single time-point; it could not capture seasonal dynamics, and the functional analysis relies on prediction rather than direct measurement. Future research that combines multi-time-point sampling with meta-transcriptomics will be essential for comprehensively revealing the spatiotemporal dynamics of microbial functions and their links to soil physicochemical properties. Additionally, while full-length sequencing significantly improves taxonomic resolution, we acknowledge the inherent amplification biases associated with universal PCR primers. Factors such as differential amplification efficiencies and sequence length polymorphisms (particularly in the fungal ITS region) may preferentially amplify certain taxa, potentially skewing relative abundance estimates. Furthermore, we acknowledge that the extremely high R^2^ values observed in our PERMANOVA analyses (0.884 for bacteria and 0.970 for fungi) are likely inflated artifacts caused by spatial autocorrelation, inherent to the localized sampling of subplots within representative fields. While these values confirm strong structural divergence driven by cropping patterns at these specific sites, the effect size should not be broadly extrapolated to the regional level.

## 5. Conclusions

This study demonstrates that distinct cropping patterns in Ningxia’s irrigation-silted soil selectively shape divergent bacterial and fungal communities. Using full-length 16S rRNA and ITS sequencing, we revealed that intensive vegetable cultivation promoted bacterial diversity and functions related to specific fertilizer inputs, but also enriched for potential fungal pathogens such as Fusarium. In contrast, maize cultivation fostered a high diversity of fungi, particularly enriching symbiotic arbuscular mycorrhizae. These findings highlight a critical trade-off between nutrient enhancement and potential pathogen risk in intensive agriculture and provide a theoretical basis for developing more sustainable soil management strategies.

Several limitations should be acknowledged. This study is based on a single time-point, it could not capture seasonal dynamics, and the functional analysis relies on prediction rather than direct measurement. Future research that combines multi-time point sampling with meta-transcriptomics will be essential to comprehensively reveal the spatiotemporal dynamics of microbial functions and their links to soil physicochemical properties.

## Figures and Tables

**Table 1:** Physicochemical properties of soils under different land-use types (mean ± SD, *n* = 3).

Soil Properties	CK Mean ± SD	CD Mean ± SD	YM Mean ± SD
pH	8.71 ± 0.04 a	8.32 ± 0.06 b	8.29 ± 0.04 b
EC (mS/cm)	24.30 ± 0.23 a	22.5 ± 0.34 b	21.8 ± 0.43 b
Organic (g/kg)	10.20 ± 1.90 c	22.00 ± 1.30 a	15.6 ± 2.40 b
Total_N (g/kg)	0.81 ± 0.05 c	1.72 ± 0.05 a	1.249 ± 0.03 b
Total_P (g/kg)	0.91 ± 0.10 c	1.60 ± 0.10 a	1.04 ± 0.10 b
Total_K (g/kg)	17.2 ± 0.17 a	15.5 ± 0.13 b	15.10 ± 0.20 b
Avail_N (mg/kg)	26.00 ± 0.22 c	163.00 ± 0.45 a	158.00 ± 0.56 b
Avail_P (mg/kg)	12.40 ± 0.37 c	162.00 ± 0.50 a	24.70 ± 0.45 b
Avail_K (mg/kg)	115.00 ± 23.96 c	620.00 ± 14.45 a	148.00 ± 10.74 b
Salt (g/kg)	3.71 ± 0.26 a	0.76 ± 0.12 b	0.82 ± 0.23 b
Moisture (%)	9.67 ± 1.98 c	75.37 ± 2.93 a	70.40 ± 3.66 b

The letters a–c explain the significant difference using statistical analysis.

## Data Availability

The raw sequencing data generated in this study have been deposited in the BioProject database of the National Genomics Data Center (NGDC) and the China National Center for Bioinformation (CNCB) under the accession number PRJCA063215.
